# Molecules and Experimental Models in Mitochondrial Disorders

**DOI:** 10.3390/life15020270

**Published:** 2025-02-11

**Authors:** Paola Loguercio Polosa, Francesco Bruni

**Affiliations:** Department of Biosciences, Biotechnologies and Environment, University of Bari ‘Aldo Moro’, 70125 Bari, Italy

The life functions of eukaryotic cells are intricately regulated by mitochondria. Despite the long-standing reputation of these organelles as the “powerhouse” of cells, decades of mitochondrial research unveiled loads of novel tasks, many of which have turned out to be unrelated to bioenergetic metabolism [1]. Therefore, the functional complexity and the multifaceted nature of mitochondria make it difficult to clearly define their cellular role [2]. The synthesis of heme, ubiquinone, lipids, amino acids, steroid hormones, and Fe–S clusters are among the many anabolic processes that take place in mitochondria. Moreover, the import of macromolecules and the exchange of metabolites, nucleotides, membrane lipids, and ions with other subcellular compartments are just a few of the many cellular activities in which these organelles take part. Remarkably, as dynamic entities going through cycles of fission and fusion, mitochondria are able to adjust to changes in metabolism or cellular stress thanks to their structural transitions [3].

The mitochondria, which descend from bacterial endosymbionts, are also unique for having retained their genome. They possess sophisticated machineries for mtDNA replication and expression that rely on proteins encoded by nuclear genes [4]. Therefore, changes in mitochondrial functions and dynamics as well as mutations in both nuclear and mitochondrial genes involved in mtDNA metabolism cause a wide range of dysfunctions and syndromes, often referred to as mitochondrial diseases. In addition, severe metabolic and age-related pathologies (obesity, diabetes, Parkinson’s disease, and many more), as well as ageing itself, have been linked to mitochondrial dysfunction [5].

The Special Issue “Advances in Mitochondrial Biology” collects research and review articles aimed at providing an updated perspective on biochemical models and molecular mechanisms underlying mitochondrial disorders.

Mitochondrial dysfunction is a key factor in many pathologies. Among these, sarcopenia is a progressive age-related condition that significantly reduces the life quality of the elderly population. Sarcopenia is characterised by muscular atrophy, which impairs muscle tissue and results in a reduction in muscle volume and total fibre number; in this condition, adipose and fibrous tissue gradually replace the muscle tissue. In their review, Jeong et al. aim to elucidate the relationship between sarcopenia and mitochondria [6]. The authors focus their attention on the (RET)-mediated regulation of mtROS production, mitochondrial biogenesis, mitochondrial dynamics, and mitophagy. In addition to the AET approach, RET is proposed as a potential therapy to improve mitochondrial function in ageing skeletal muscles and treat sarcopenia.

Another pathological condition linked to mitochondria malfunction is the cardiovascular disease. In CVD, cardiomyocyte mitochondria exhibit deficiencies in ATP synthesis and calcium regulation and accumulate oxidatively damaged debris. Reiter and colleagues review the beneficial effect of melatonin in cardiac pathological conditions [7]. Melatonin is a multifaceted molecule proved to be protective against CVD and cardiac damage at both systemic and cellular levels. It is synthesised and found at high levels in mitochondria, which are the main sites of ROS production and important regulators of calcium homeostasis. Interestingly, the mitochondrial capacity to produce this molecule would have been acquired by eukaryotic cells about 2.5 billion years ago, in line with the endosymbiotic hypothesis. The ability of melatonin to neutralise destructive mtROS (e.g., free radicals) and its inflammatory–inhibitory effect seem to be crucial to modulating the atherosclerotic plaque initiation and development; this regulation would prevent the onset of processes that cause different subtypes of CVD. The authors emphasise that the non-toxic nature of melatonin is supported by experimental data and propose that its beneficial action on cardiac pathophysiology should be exploited through clinical trials in humans.

A relevant feature of mitochondrial physiopathology is its correlation with cancer development. Czegle et al. present a comprehensive overview of mitochondria-driven tumour growth in breast, endometrial, and ovarian cancers [8]. The review focuses on describing the genetic background for each of these three malignancies, outlining all the genes whose alterations are associated with tumorigenesis. In detail, the authors highlight how these genes affect both biochemical and molecular aspects of mitochondria, such as mitochondrial glucose and fatty acid metabolism, ROS generation, mitochondrial biogenesis and dynamics, mitophagy, and mtDNA transcription. After discussing the similarities and differences among the reviewed cancers, they draw the conclusion that targetable metabolic and mitochondrial alterations may be helpful in the fight against therapy-resistant malignancies.

In addition to the reviews mentioned above, this Special Issue includes research articles describing the use of three different experimental models of mitochondria-associated diseases. The first paper comes from our group and examines the RNA19 in MELAS trans-mitochondrial cybrids carrying the mtDNA 3243A > G transition [9]. RNA19 is a long unprocessed mitochondrial transcript, unusually stable. According to our analyses, RNA19 accumulates in the mutant cell line preferentially associating with large protein complexes, very likely the mt-LSU. Previously, we demonstrated that the isolated LARS2 C-terminal domain (Cterm) was able to bind RNA19 and recover the mutant phenotype in MELAS cells. In the current work, exogenous expression of the Cterm peptide lowers the capacity of RNA19 to co-sediment with mt-LSU, likely by sequestering the RNA molecule prior to its accumulation on the mitoribosome. Overall, mitochondrial RNA19 might mediate the rescue activity of the Cterm and could be a potential regulatory molecule in both physiological and pathological conditions.

Another study, based on an *E. coli* model system, was conducted by Vik’s research group with the aim of rapidly analysing the impact of mutations of the human mitochondrial gene *ND1* [10]. Several clinical mutations of the human *ND1* have been modelled in the gene *NuoH* of bacterial Complex I. The authors have identified nine mutations that are known to be pathogenic and assessed the effect of the amino acid substitutions on *E. coli* Complex I activity. The outcomes of this experimental model are in line with the pathogenicity that was already demonstrated in humans. Therefore, despite the lack of positive controls (benign mitochondrial mutations), this study shows that a bacterial model system could provide valuable information regarding mutations in mitochondrial genes, confirming the pathogenicity of both known variants and those yet to be determined.

In the last research article, Tomczewski et al. investigate the potential role of the mitochondrial enzyme LPAATδ/AGPAT4 in energy metabolism [11]. To this aim, a cohort of fourteen-month-old *Lpaatδ* knockout mice was generated and analysed. In particular, body weight, food intake, locomotion, and several metabolic parameters (oxygen consumption, carbon dioxide production, respiratory exchange ratio, and total energy expenditure) were measured in mutant and control mice. *Lpaatδ*^−/−^ mice had higher values of metabolic parameters than wild-type littermate mice, while they showed significantly lower mean body weights compared to controls. The LPAATs/AGPATs enzyme family is involved in phospholipid metabolism, catalysing the formation of phosphatidic acid from lysophosphatidic acid. Therefore, according to the authors’ hypothesis, reduced body weights and increased energy expenditure recorded in the middle-aged *Lpaatδ*^−/−^ mice model may be linked to the lack of LPAATδ-derived phosphatidic acid, which could be relevant to mitochondrial functionality (e.g., the phosphatidic acid role in coordinating mitochondrial dynamics).

In conclusion, the set of articles included in this Special Issue emphasises the importance of characterising individual molecules involved in mitochondrial metabolism as well as the centrality of experimental model systems in the study of mitochondrial disorders.

## Abbreviations

The following abbreviations are used in this manuscript:

mtDNA	mitochondrial DNA
RET	Resistance Exercise Training
AET	Aerobic Exercise Training
mtROS	mitochondrial reactive oxygen species
CVD	cardiovascular disease
ATP	adenosine triphosphate
MELAS	Mitochondrial Encephalopathy with Lactic Acidosis and Stroke-like episodes
mt-LSU	mitoribosomal large subunit
LARS2	leucyl-tRNA synthetase 2
LPAAT	lysophosphatidic acid acyltransferase
AGPAT	acylglycerophosphate acyltransferase
